# A complementary learning systems model of how sleep moderates retrieval practice effects

**DOI:** 10.3758/s13423-024-02489-1

**Published:** 2024-03-26

**Authors:** Xiaonan L. Liu, Charan Ranganath, Randall C. O’Reilly

**Affiliations:** 1 Department of Psychology, The Chinese University of Hong Kong, Hong Kong, Hong Kong; 2 Department of Psychology, University of California, Davis, CA USA; 3 Center for Neuroscience, University of California, Davis, CA USA; 4 Department of Computer Science, University of California, Davis, CA USA

**Keywords:** Testing effect, Sleep, Consolidation, Retrieval practice, Complementary learning systems

## Abstract

While many theories assume that sleep is critical in stabilizing and strengthening memories, our recent behavioral study (Liu & Ranganath, 2021, *Psychonomic Bulletin & Review*, *28*[6], 2035–2044) suggests that sleep does not simply stabilize memories. Instead, it plays a more complex role, integrating information across two temporally distinct learning episodes. In the current study, we simulated the results of Liu and Ranganath (2021) using our biologically plausible computational model, TEACH, developed based on the complementary learning systems (CLS) framework. Our model suggests that when memories are activated during sleep, the reduced influence of temporal context establishes connections across temporally separated events through mutual training between the hippocampus and neocortex. In addition to providing a compelling mechanistic explanation for the selective effect of sleep, this model offers new examples of the diverse ways in which the cortex and hippocampus can interact during learning.

## Introduction

In the field of learning and memory, many theories assume that sleep plays a critical role in stabilizing or strengthening memories for events, such that they are resistant to interference. More detailed theories, such as the active systems consolidation theory, propose that neural representations of recent events are spontaneously activated (i.e., “sleep replay”) during slow-wave sleep (SWS), thereby strengthening these representations and improving retention (Diekelmann & Born, 2010; Geva-Sagiv & Nir, 2019; Klinzing et al., 2019; Tamminen et al., 2013; Tamminga et al., 2010). However, we also know that repeatedly reactivating memories during wake can enhance the retention of recently learned information, independent of sleep. These findings raise the question of whether sleep confers unique benefits or is simply the same as what happens when we repeatedly access memory while awake (Antony et al., 2017)

In this paper, we explore the possibility that sleep does play a special role, enabling the brain to discover connections between events that occurred at different times. Using a biologically based computational model of cortico–hippocampal interactions, based on the complementary learning systems (CLS) framework (O’Reilly et al., 2014), we modeled behavioral results showing that events that are initially learned across longer temporal delays can become integrated during sleep. Our model suggests that when memories are activated during sleep, the reduced influence of temporal context establishes connections across temporally separated events through mutual training between the hippocampus and neocortex. Below, we describe how the model helps to bridge the gap between the effects of sleep on memory and the effects of reactivating memories during wake.

### The Selective Effects of Sleep on Memory

Although the active systems consolidation theory predicts that memory reactivation during SWS should promote memory consolidation, available evidence does not consistently support the idea. Behavioral effects of sleep-mediated consolidation have been inconsistent across studies and paradigm dependent (Cordi & Rasch, 2021). For example, some studies showed that sleep could protect associative memories against interference (Ellenbogen et al., 2006, 2009), two recent studies failed to replicate this finding (Bailes et al., 2020; Pöhlchen & Schönauer, 2020).

Rather than strengthening all memories or slowing forgetting, sleep is more likely to have selective effects. For example, some studies have found that sleep improved memory for shared properties of newly learned semantic categories (Schapiro et al., 2017) and facilitated the incorporation of new information into existing semantic knowledge (Tamminen et al., 2013). Other studies suggest that sleep selectively facilitates weakly learned information (e.g., Schapiro et al., 2018). These findings suggest that SWS does not simply stabilize memories, and instead, it might play a more complex role, reorganizing representations of past experience (Lewis & Durrant, 2011; Singh et al., 2022).

### Empirical results that challenge the active systems consolidation theory

**Fig. 1:**
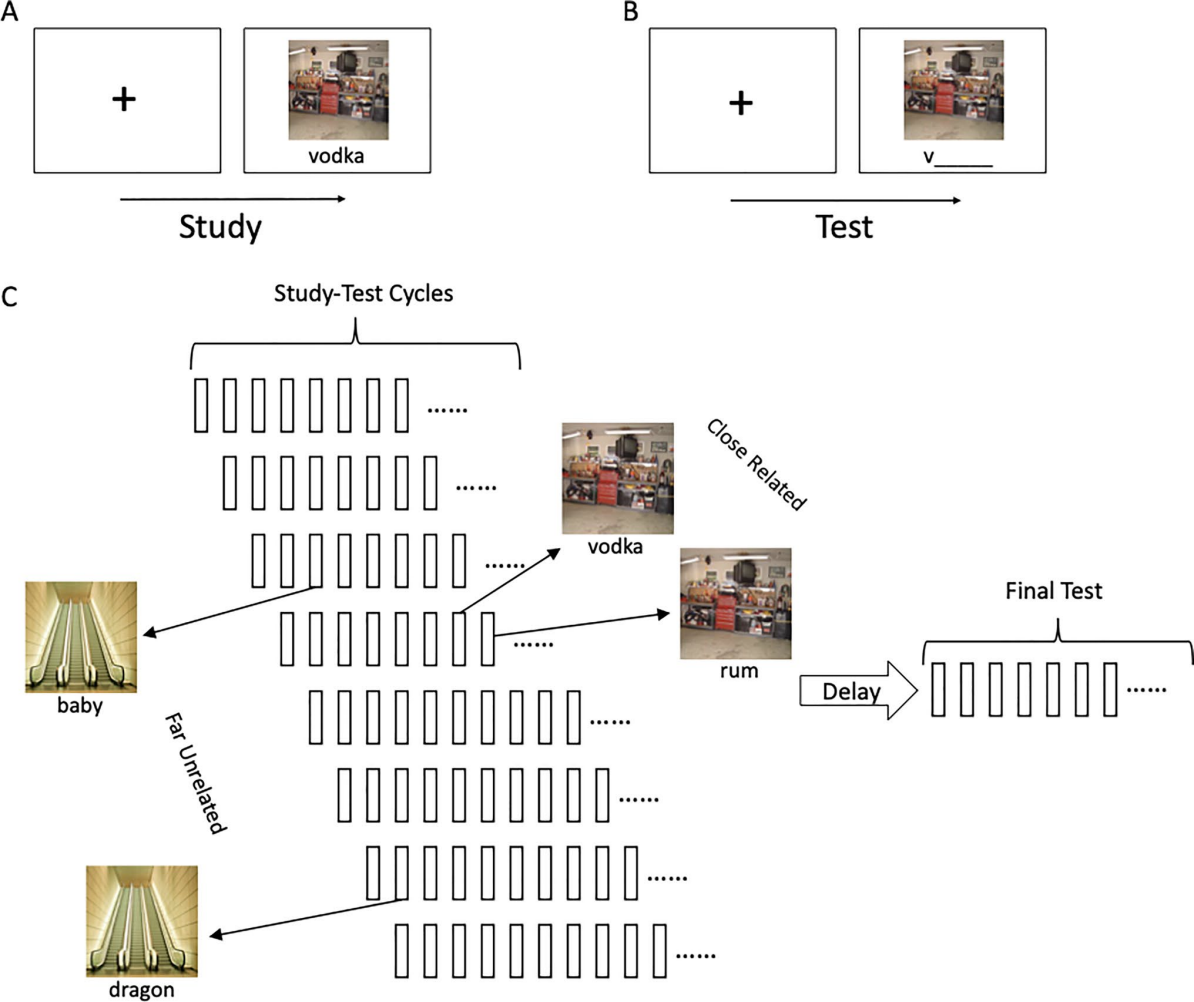
Paradigm of Liu and Ranganath (2021). **A** Illustration of study procedure. **B** Illustration of test procedure during retrieval practice and the final test. **C** Illustration of the overall experimental paradigm. The delay was manipulated between-subject as wake versus sleep

Here, we simulate recent evidence that raises an important challenge for theories proposing that memory consolidation is mediated by simple effects of memory reactivation during SWS (Liu & Ranganath, 2021). It is well known that retrieval of a target item during waking states can affect the retention of items that are not retrieved, facilitating retention of items that are well integrated with the target and causing forgetting of items that compete with the target. We recently examined how these “spillover” effects of retrieval practice might interact with sleep-mediated consolidation in three different experiments that varied several factors (Liu & Ranganath, 2021). Specifically, subjects were asked to learn pairs of scene–word associations, with each scene linked to two distinct words (pairmates) that shared either a semantic connection or not. In addition, the experiments systematically manipulated the temporal proximity of these pairs to determine the impact of retrieval on their retention (Fig. 1).

**Fig. 2:**
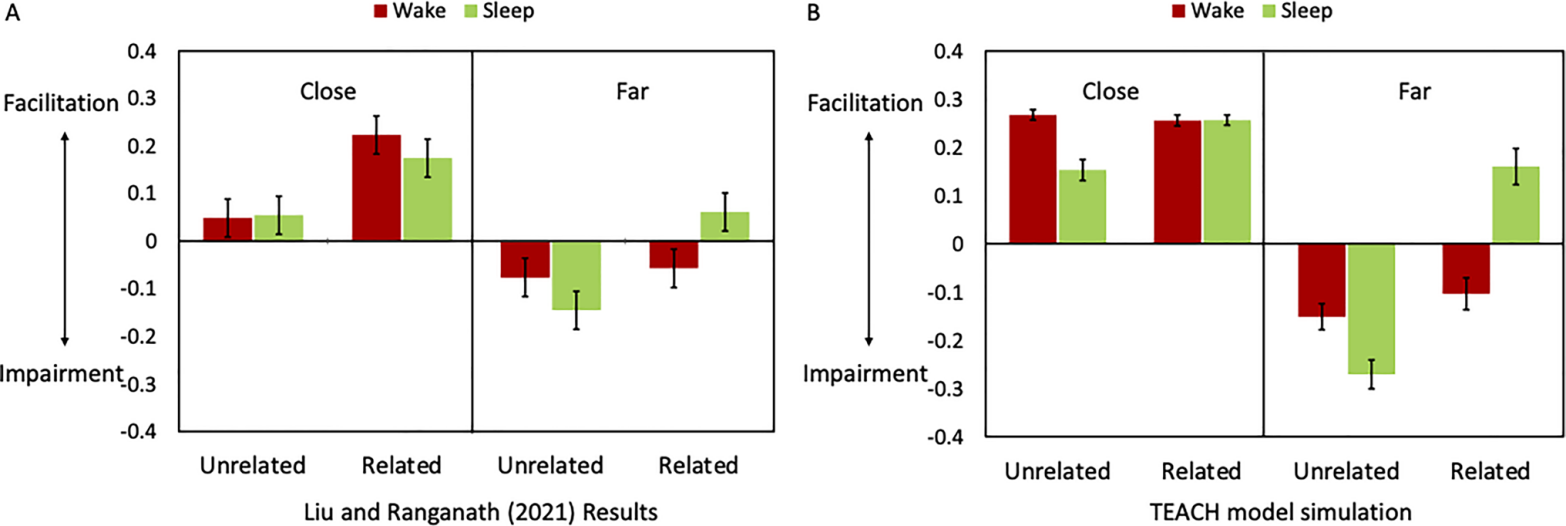
**A** Behavioral results of Liu and Ranganath (2021). **B** Simulation results of the TEACH model. Graph shows mean final test recall differences between nontarget and control trials separately as a function of temporal proximity (close vs. far) and semantic relatedness. Error bars denote 95% confidence intervals

We (Liu & Ranganath, 2021) did not find a global effect of sleep on retention, nor did we find that sleep generally increased or reduced the effects of retrieval practice on unstudied items (Fig. 2). For instance, regardless of subjects’ sleep status, retrieval of a specific scene–word pair invariably improved the recall of “temporally close” pairmates—those studied within the same temporal context (i.e., retrieval-induced facilitation, RIFA, e.g., Chan, 2009). In contrast, recalling a target led to decreased retention of “temporally far” pairmates—those from different lists and semantically unrelated to the target (i.e., retrieval-induced forgetting, RIF, e.g., Anderson & McCulloch, 2003). Critically, only one of our findings was directly affected by sleep: Retrieval practice impaired retention of temporally far, semantically related pairs if subjects were unable to sleep during the retention interval, but it facilitated retention of these items if subjects were able to sleep. In other words, sleep reversed the negative effects of retrieval practice, enabling participants to “rescue” memories that might otherwise be lost due to interference.

The results of Liu and Ranganath (2021) are, to some extent, consistent with the complementary learning systems (CLS) framework (McClelland et al., 1995; O’Reilly et al., 2014). According to CLS, the hippocampus enables fast learning of specific pieces of information (i.e., episodic-like memory), whereas the neocortex supports slow learning that supports generalization (i.e., semantic memory). In this framework, reactivation of hippocampal representations during sleep enables the hippocampus to “teach” the neocortex such that new information can be incorporated without disrupting preexisting neocortical representations (McClelland et al., 1995; O’Reilly et al., 2014).

Singh et al. (2022) have elaborated on the CLS framework with their C-HORSE (complementary hippocampal operations for representing statistics and episodes) model. According to this model, during SWS, the hippocampus is instrumental in constructing neocortical representations of new information via hippocampal–cortical replay. The model has been notably successful in explaining empirical observations that sleep enhances the learning of semantic category structures, thereby augmenting neocortical knowledge (e.g., Schapiro et al., 2017).

This framework fits with Liu and Ranganath’s (2021) finding that sleep uniquely enabled subjects to integrate information across temporally distinct but related learning episodes. However, in the original framework, like the active systems consolidation hypothesis, consolidation was simply envisioned as an effect of reactivating hippocampal memories during sleep. As such, it cannot fully account for the different effects of retrieval practice during wakefulness and sleep.

### The current model

Here, we sought to gain traction on how sleep-mediated consolidation could produce such a specific effect on memory that was qualitatively different from the effects of awake memory retrieval. We propose a novel extension of the biologically-based CLS model of hippocampal function (O’Reilly et al., 2014), referred to as the TEACH (TEsting Activated Cortico-Hippocampal interaction).

Consistent with the CLS and Antony et al. (2017), the model assumes that, during waking, hippocampal memories are retrieved through a combination of item and temporal context cues and that the hippocampus trains the neocortex during memory retrieval (e.g., Antony et al., 2017; Ferreira et al., 2019; Ritvo et al., 2019; see Liu et al., 2021 for a review). During waking retrieval practice, temporal context acts as a barrier, such that reactivation of one memory in the hippocampus will not lead to retrieval of related memories learned in different contexts.

To account for the effects of sleep, we start with the assumption that underlies active systems consolidation theory: the hippocampus interacts with the neocortex during SWS (Singh et al., 2022). In addition to assuming the hippocampus trains the neocortex, our model incorporates both directions of cortico-hippocampal interactions. Specifically, following recent empirical findings (Rothschild et al., 2017), the model assumes that, during sleep, the reactivation of associations starts in the neocortex. Because the weights of temporal context are weakened during sleep (Schechtman et al., 2023a, 2023b), semantic connections in the neocortex provide a way in which the cortex effectively trains the hippocampus to learn about these connections. We also assume that recently activated associations, such as those experienced during retrieval practice, are prioritized for replay during sleep. This assumption is based on the empirical finding (Liu & Ranganath, 2021) that RIF transitions to RIFA following a period of sleep. This pattern suggests that retrieval practice moderates the effect of sleep on the untested association. Specifically, if sleep replay were uniform for both retrieval-practiced items and control items (which did not undergo retrieval practice), the benefits of sleep would be similarly distributed across these groups, and the observed switch from RIF to RIFA would likely not occur. We will discuss how these assumptions are congruent with the literature in the General Discussion.

In the remainder of the paper, we first present the methods, including a high-level summary of the principles and mechanisms in our computational model, followed by our simulation results of the Liu and Ranganath (2021) data. Then, we discuss connections to other related computational and theoretical work and implications for future work, including further testable predictions of our framework.

## Methods

### Overview of the model

**Fig. 3:**
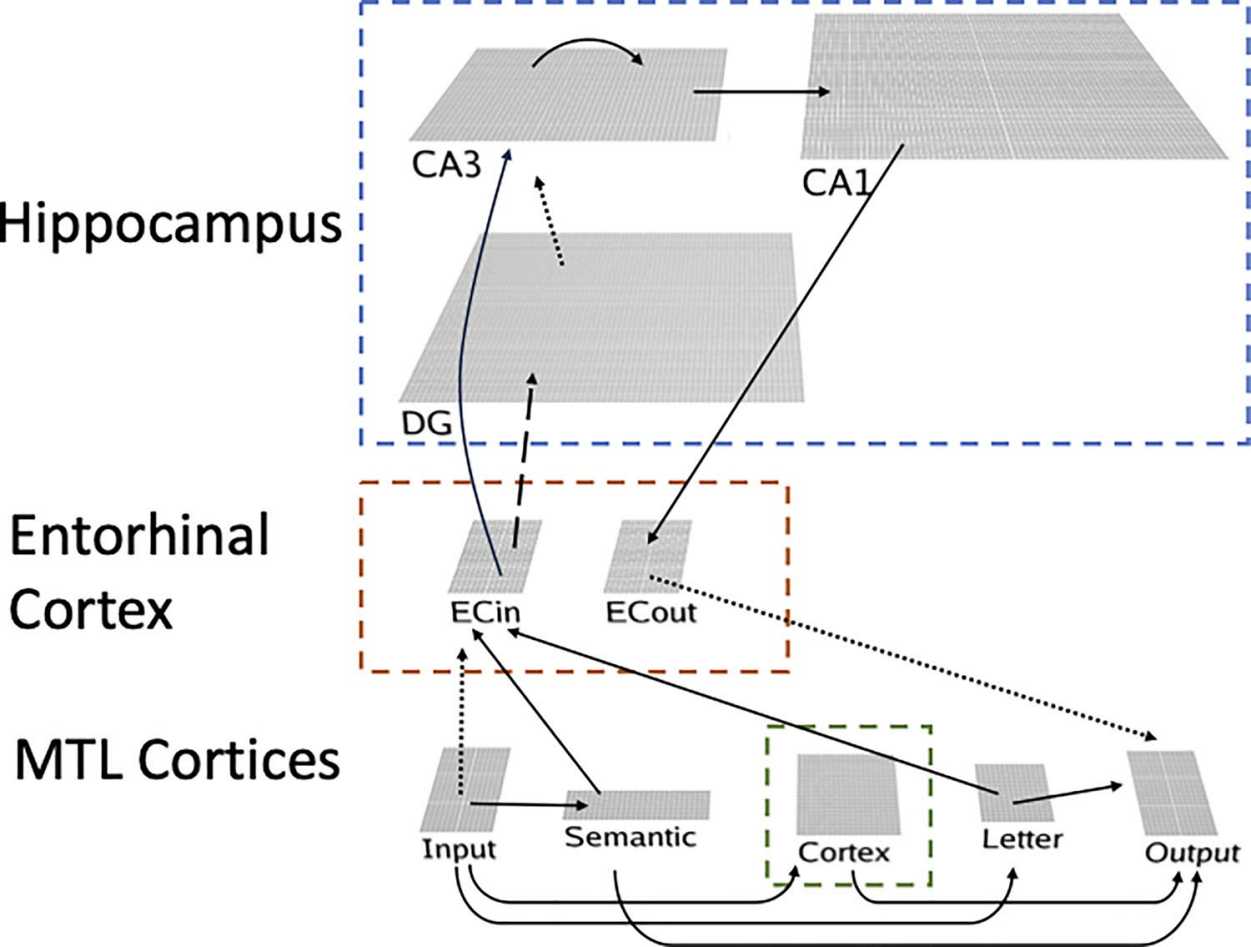
Overview of the TEACH model architecture. Solid lines represent projections that have error-driven learning + Hebbian learning; dashed lines represent projections that only have Hebbian learning, and dotted lines represent projections that do not learn in the model

The model simulates both the hippocampal and neocortical systems (Fig. 3), and the two learning systems are bidirectionally connected, and we assume that the hippocampal and neocortical systems jointly contribute to memory retrieval, consistent with the CLS framework (O’Reilly et al., 2014). Specifically, retrieval of a specific item can be seen as falling on a continuum, with varying degrees of dependence on the two learning systems depending on the task, item, and other variables.

One essential principle of the CLS framework is that the hippocampus is capable of rapid learning, whereas neocortical areas exhibit slow learning rates, and that cortico–hippocampal interactions can speed up the process of cortical learning, such that new information can be incorporated without disrupting pre-existing representations (McClelland et al., 1995; O’Reilly et al., 2014). These interactions could occur when memories are recalled during the waking state, or when recently learned information is reactivated by the hippocampus during sleep. This idea is consistent with recent theories (e.g., Antony et al., 2017; Ferreira et al., 2019; Liu et al., 2021; Ritvo et al., 2019) suggesting that online reactivation (such as retrieval) of recently acquired memories in the hippocampus can drive learning in the neocortex. Other recent studies have shown that communication between the hippocampus and neocortex during sleep can be initiated in the neocortex, and there is a cortical–hippocampal–cortical loop of information flow during sleep (Rothschild et al., 2017).

The model was implemented using the Leabra framework (O’Reilly et al., 2015; O’Reilly & Rudy, 2000), which supports both cortical and hippocampal systems within one overall model. The hippocampal system has recently been updated with a more complete implementation of error-driven learning mechanisms in areas CA1 and CA3 (Zheng et al., 2022), building on earlier work (Ketz et al., 2013).

Learning in neural networks occurs by modifying synaptic weights between sending and receiving neurons. The Leabra framework is based on the combined contributions of two distinct, widely used learning rules: Hebbian and error driven. Hebbian learning posits that synaptic weights are strengthened when they are co-activated (“cells that fire together, wire together”; Hebb, 2005), and it serves as a kind of heuristic bias toward encoding statistical correlations in the Leabra model. Error-driven learning provides the most important form of learning, by adjusting weights to minimize errors in a network’s performance, as in the widely used error backpropagation algorithm (Rumelhart et al., 1986). Leabra uses a biologically plausible form of error-driven learning that leverages bidirectional connectivity to communicate error signals in the same way that any other neural activity is communicated, with synaptic learning sensitive to two different phases of activity over time that represent an expectation followed by an outcome or target state (O’Reilly, 1996; O’Reilly & Rudy, 2000).

To directly examine the necessity of the hypotheses, we simulated three alternative models of sleep—namely, the Sleep-PriWeak model, in which nontested and pairs were prioritized for replay, the Sleep-All model, in which all pairs were equivalently replayed, and the Sleep-AddRP model, in which sleep was equivalent to additional retrieval practice.

### The neocortical system

The neocortex supports basic sensory-motor processing and higher-level semantic and association knowledge, captured by the input, output, letter, semantic, and cortex layers in our model. We simplify the model by driving the input and output layers with fixed patterns of the task events, containing information about scene–word pairs and temporal context (as random vectors). These vectors are represented as six pools of 49 neurons (as a 7 × 7 matrix). One pool represents the scene cue, two pools represent the two words that are associated with the scene, one pool represents the first letters of the words, which also serve as cues at test, and the remaining two pools represent the temporal context. Each input pattern was constructed by randomly assigning 20% neurons as active and the rest of the neurons as inactive.

The relevant long-term semantic knowledge for spelling and semantic associations of the items used in the study are separately encoded in the letter and semantic layers. The letter layer receives information from the letter pool of the input layer and encodes the orthographic spelling of the words, sending its output to the word pools of the output and the hippocampal system. The semantic layer receives information from each of the word pools of the input layer and encodes semantic representations of the words, sending its output to both word pools of the output and the hippocampal system. Activation of a word will partially activate semantically related words through the Semantic layer. The Semantic layers are pre-trained before learning the scene–word associations. The contribution of semantic to the hippocampal system is inhibited by the temporal context input, capturing a hypothesized controlled task-based top-down biasing function of the form typically attributed to the prefrontal cortex (Miller & Cohen, 2001).

We assume that the novel scene–word associations that are established within the context of the task are learned most strongly in the neocortex by the MTL cortical areas surrounding the hippocampal formation, including the perirhinal cortex, which has been implicated in higher-order associative learning of this form (Inhoff & Ranganath, 2017; Ranganath & Ritchey, 2012; Ritchey et al., 2015). In the model, this occurs in the connections from the scene and word pools of the input layer to the cortex layer, which then drives corresponding pools of the output layer that represents the relevant output signal. Biologically, this output signal is likely conveyed by deep-layer neurons and their projections, which exist in every neocortical area. The Output layer also receives information from the output of the hippocampus, via its entorhinal cortex output layer. According to the CLS framework, the cortical layers use a slower learning rate and learn more overlapping representations than the hippocampus. Therefore, during initial learning, the difference between the fast-learning hippocampal and slower-learning cortical representations generates error signals that drive learning in these neocortical pathways.

### The hippocampal system

The hippocampal system in our model receives cortical inputs into its entorhinal cortex input layer (ECin, representing the superficial layers of EC), which then projects to both the DG and area CA1 in the hippocampus. For simplicity, the ECin just mirrors the same pool structure as the Input layer. The DG layer is larger than ECin and features high levels of inhibition, resulting in only very sparse representations that functionally separate the patterns of highly similar inputs from ECin, which then drive the CA3, which also receives direct ECin projections. Our recent model of error-driven learning in the hippocampus shows how the DG input to CA3 can drive error-driven learning relative to the direct ECin pathway inputs (Zheng et al., 2022). Consistent with the classic Hebb–Marr model of the hippocampus, the recurrent connections among CA3 neurons support pattern completion, where an activated representation can retrieve its previously learned associations. The CA3 then drives the CA1, which functions as a sparse auto-encoder for all information in the EC input/output pathway, activating any retrieved memory elements onto the ECout (deep entorhinal layers).

Note that there are two separable hippocampal loops: a monosynaptic pathway (containing only a single synapse within the hippocampus (from ECin → CA1→ ECout), and a trisynaptic pathway (from ECin→ DG → CA3→ CA1→ ECout).

The network size parameters of this model are summarized in Table 1.

**Table 1:** Parameters for network sizes

**Network layer**	**Size**
Input pool size	7 × 7
Input number of pools	2 × 3
ECin pool size	7 × 7
ECin number of pools	2 × 3
ECout pool size	7 × 7
ECout number of pools	2 × 3
DG size	70 × 70
CA3 size	50 × 50
CA1 pool size	30 × 35
CA1 number of pools	2 × 3
Letter size	14 × 14
Semantic size	14 × 14
Cortex size	20 × 20

The numbers for pool sizes indicate the number of neurons in each specific pool.

### Model training

The main training involved 12 unique scene patterns, with each scene paired with two word patterns or pairmates, resulting in 24 scene–word associations and 12 groups of pairmates sharing the same scene. The factorial experimental design incorporated four factors: retrieval practice, temporal distance, semantic relatedness, and sleep. The retrieval practice manipulation resulted in three types of trials: For some of the scene–word associations, one pairmate, the retrieval target, was retrieval practiced after the initial study. We refer to the nonpracticed pairmate as a nontarget. Finally, for control associations, neither of the pairmates were practiced.

The temporal distance manipulation focused on the distance between the practiced scene–item association and the unpracticed pairmate. Unpracticed pairmates were either adjacent (i.e., the two associations were presented with the same temporal context) and far (i.e., the two associations were studied with different temporal contexts). In the far condition, the nontarget was always studied in an earlier list than the target to ensure that participants learned both associations before retrieval practice. The semantic relatedness factor was manipulated by whether the Semantic layer was pre-trained, that is, whether activation of one pairmate could also activate the other pairmate. Finally, as described in more detail below, the sleep factor was manipulated by whether the sleep training procedure was given. Training in all the conditions used the same set of 32 associations, and after training and testing of each condition, the model was reinitialized with the same random seeds. We trained the model with 20 independent runs with a new random seed used for each run.

#### Pretraining

During pretraining of the semantic relationships, each word was presented to the input layer. In the related condition, both the same word and the related word used in model training were presented to the ECin layer as the target pattern, to drive error-driven learning of the semantic relationships. In the unrelated condition, the same word and an unused random word pattern were presented to the ECin layer. To ensure strong semantic relationships, pretraining of each word was repeated for 30 times (“epochs”).

#### Initial study

During the initial study phase, the scene–word associations were presented to the input layer in succession. In the close condition, the two pairmates associated with one scene were trained next to each other, and all the associations were trained with the same temporal context pattern. In the far condition, one pairmate associated with each scene was trained with the first temporal context pattern, and the second pairmates were trained with a different temporal context pattern. During each initial study trial, the correct input pattern from the ECin layer directly drives a corresponding pattern over the ECout layer at the end of the theta cycle, to drive error-driven learning in the hippocampus (Ketz et al., 2013; Zheng et al., 2022).

#### Retrieval practice

In conditions involving retrieval practice, the initial study phase was followed by the retrieval practice phase, in which half of the associations were tested. During a retrieval practice trial, only the scene was presented to the input layer along with the first letter and the temporal context. The model was supposed to complete the missing target pattern in the ECout layer through the hippocampal learning system and in the CORout layer through the neocortical learning system. Unlike the initial study phase, the correct pattern was not provided to the model, consistent with the behavioral experiment (Liu & Ranganath, 2021) in which no feedback was provided. The ECout pattern served as the target for the CORout layer, and the difference between the two layers created the error signal that trained the neocortical system.

#### Sleep

In conditions involving sleep, the sleep procedure was simulated after the retrieval practice phase. During sleep, the scene–word associations were presented to the input layer without temporal context. As introduced earlier, the relative weight of semantic input to ECin was stronger when the temporal context input was shut off. Thus, input words could also activate semantically related words in ECin. Similar to the initial study phase, the ECin pattern drives ECout at the end of the theta cycle, driving error-driven learning. Following the assumption that retrieval practiced associations are prioritized to replay during sleep, after training of all associations, retrieval practiced associations were trained for additional epochs. Different training rules were applied for the alternative models. Specifically, for the Sleep-PriWeak model, associations that were not retrieval practiced received additional training epochs; for the Sleep-All model, all associations were trained for the same number of epochs; for the Sleep-AddRP model, the sleep session was identical to the retrieval practice session.

### Model testing

During the final test, weight updating (i.e., learning) was disabled. Only the scene and first letter were presented for each final test trial. This setup was designed to mimic the elapsed time between the initial learning phase and the final test, effectively removing the immediate study context to simulate the delay. This approach aligns with the procedure used by Antony et al. (2022), where a similar methodology was employed to understand the effects of context change over time on memory retrieval. It is important to note that the inputs during the final test were kept consistent across both the wake and sleep conditions to ensure that any observed differences in performance could be attributed to the state-dependent processes rather than the passage of time alone.

The model performance was evaluated by the *d′* of the output layer, defined as *d′* = *Z*(hit rate) − *Z*(false-alarm rate), where the hit rate was the proportion of active units in the original target that was also active in the retrieved pattern of the output layer, and the false-alarm rate was the proportion of inactive units that were active in the output layer. This method of performance evaluation allows us to capture the precision of memory retrieval, without the imposition of an arbitrary accuracy threshold that might otherwise be necessary for using mean trial accuracy in model simulations.

## Results

Following the behavioral study (Liu & Ranganath, 2021), our primary analyses focused on *d′* differences between the nontarget and control trials on the final test. The behavioral results of accuracy difference between nontarget and control trials showed a three-way interaction between temporal distance, semantic relatedness, and sleep. Specifically, retrieval practice facilitated retention of temporally adjacent nontargets and impaired recall of temporally far and unrelated nontargets regardless of sleep, retrieval practice impaired recall of temporally far and related nontargets but retrieval practice facilitated retention of these items after postlearning sleep.

### TEACH model results

**Fig. 4:**
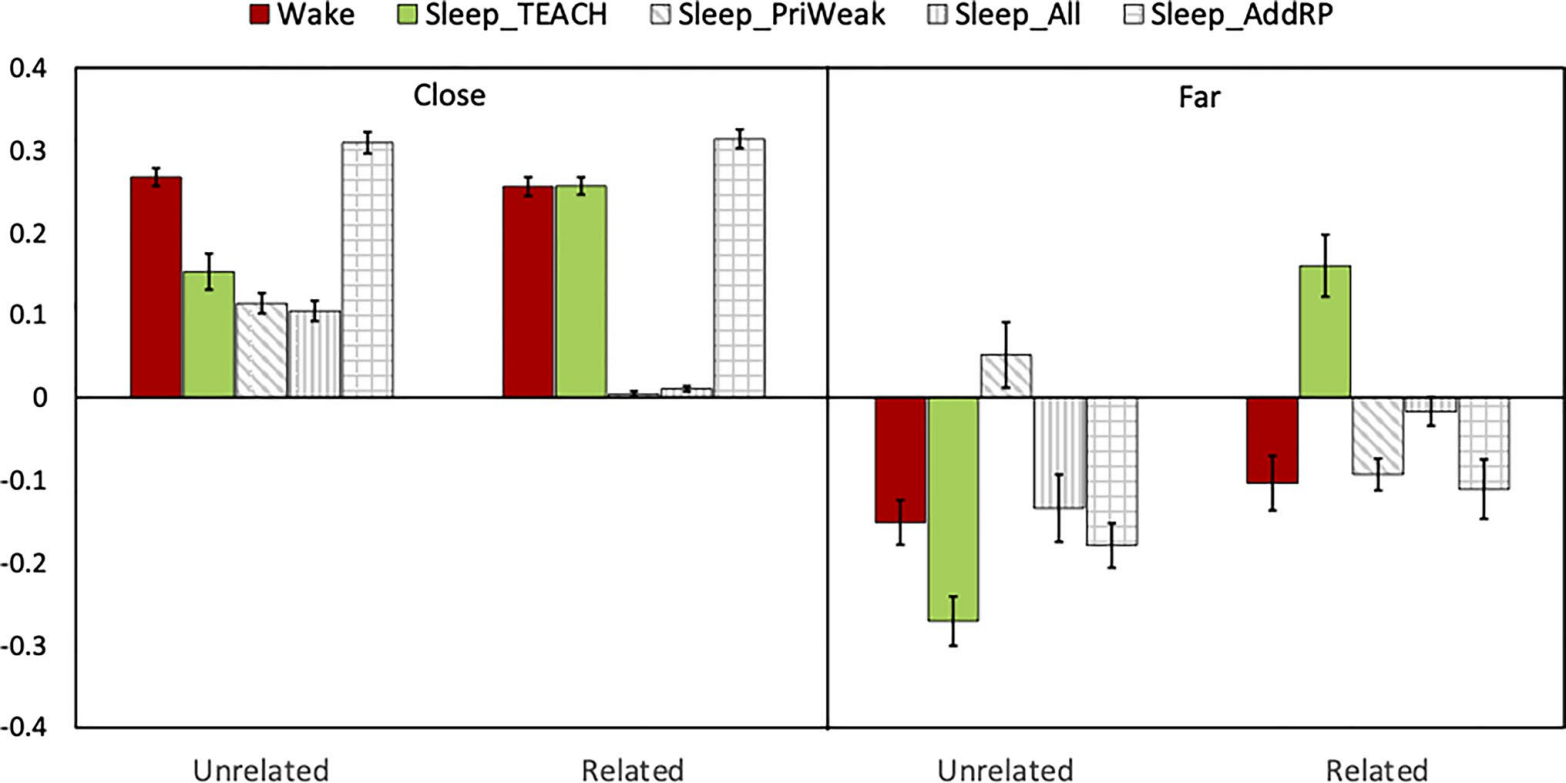
Results of the TEACH and alternative models

Simulations with the TEACH model replicated the entire pattern of results summarized above. Figures 2 and 4 show the results of the model simulation. A 2 (temporal distance: adjacent, far) × 2 (semantic relatedness) × 2 (sleep vs. wake) analysis of variance (ANOVA) revealed a three-way interaction, *F*(1, 38) = 20.21, *p* < .001, η_p_^2^ = .35. To break down this complex pattern of results, planned comparisons were conducted to examine whether retrieval practice facilitated or impaired recall of the nontarget trials compared with the control trials in each condition.

Consistent with the behavioral results, regardless of sleep and semantic relatedness, retrieval practice facilitated retention for temporally adjacent nontargets. Specifically, the recall performance for nontargets was significantly better than that for control trials in each of the temporally adjacent conditions—related/sleep: *t*(19) = 24.21, *p* < .001, *d* = 5.41; unrelated/sleep: *t*(19) = 6.99, *p* < .001, *d* = 1.56; related/wake: *t*(19) = 22.43, *p* < .001, *d* = 5.02; unrelated/wake: *t*(19) = 24.71, *p* < .001, *d* = 5.53.

Regardless of sleep, retrieval practice impaired recall of temporally far and unrelated nontargets. Specifically, the recall performance for nontargets was significantly better than that for control trials in far and unrelated conditions—sleep: *t*(19) = 9.05, *p* < .001, *d* = 2.02; wake: *t*(19) = 5.57, *p* < .001, *d* = 1.25.

Without sleep, retrieval practice impaired recall of temporally far and related nontargets, but retrieval practice facilitated retention of these items after post-learning sleep. Recall performance for nontarget was better than that for control trials in the far and related condition with sleep, *t*(19) = 4.25, *p* < .001, *d* = .95, but worse than the for control trials without sleep, *t*(19) = 3.13, *p* = .006, *d* = .70.

### Alternative model results

Our next simulations considered the viability of alternative assumptions that might explain the results of Liu and Ranganath (2021). First, we considered the possibility that sleep replay might be qualitatively similar to retrieval practice. To simulate the results under this assumption, we ran a version of the model (Sleep-AddRP) in which sleep was identical to additional retrieval practice epochs. While the Sleep-AddRP model also showed facilitation effects in temporally adjacent conditions—related: *t*(19) = 26.90, *p* < .001, *d* = 6.01; unrelated: *t*(19) = 23.11, *p* < .001, *d* = 5.17, and impairment in the far and unrelated condition, *t*(19) = 6.46, *p* < .001, *d* = 1.45, different from the behavioral results (Liu et at., 2021), it showed impairment, instead of facilitation in the far and related condition, *t*(19) = 2.99, *p* = .007, *d* = .67.

Next, we considered alternative assumptions about the prioritization of specific associations to be replayed during sleep. For example, one possibility is that, rather than prioritizing strongly learned associations, weakly learned associations might be disproportionately reactivated during sleep. Therefore, we ran a version of the model (Sleep-PriWeak) in which associations that were not retrieval practiced (including both nontargets and control trials) received additional training epochs during sleep. Consist with the TEACH model, the Sleep-PriWeak model showed facilitation in the close and unrelated condition, *t*(19) = 3.13, *p* = .006, *d* = .70. However, this model also did not capture the key finding that retrieval practice facilitated far and related nontargets after sleep. Instead, it showed impairment, *t*(19) = 4.69, *p* < .001, *d* = 1.05. There were no significant effects found in the close and related, *t*(19) = 1.23, *p* = .23, *d* = .28, and the far and unrelated, *t*(19) = 1.29, *p* = .22, *d* = .28, conditions.

Finally, we considered the possibility that replay is completely random, such that any association might be equally likely to be replayed. In this simulation (Sleep-All), all associations received the same number of training epochs during sleep. Similar to other alternative models, the Sleep-All model did not show a facilitation effect in the far and related condition, *t*(19) = .97, *p* = .35, *d* = .22. The results of the other three conditions were consistent with the TEACH model. Specifically, retrieval practice facilitated the recall of nontargets in the in temporally adjacent conditions—related: *t*(19) = 3.17, *p* = .005, *d* = .71; unrelated: *t*(19) = 8.32, *p* < .001, *d* = 1.86, and impaired recall in the far and unrelated condition, *t*(19) = 3.19, *p* = .005, *d* = .71.

## General discussion

Substantial evidence suggests that retrieving recently learned information can significantly improve retention of the reactivated information. Although evidence also suggests that memories of recent experiences might be reactivated during sleep, the effects of sleep on retention are not equivalent to the effects of retrieval practice (Liu & Ranganath, 2021). Here, we show that a biologically inspired model of cortico–hippocampal interactions can account for the effects of sleep and retrieval practice on integrating semantically related information. This model captures the key results of Liu and Ranganath (2021), which showed that retrieval practice could facilitate the retention of untested semantically related information if subjects had the opportunity to sleep between retrieval practice and the final test, even if the retrieval practice target and nontarget were learned across two temporally distinct episodes. It is noteworthy that these results cannot be explained by the idea that sleep is equivalent to retrieval practice, as shown in the Sleep-AddRP model. Instead, our modeling suggests that the reduced influence of temporal context on memory activation during sleep can account for the complex effects of sleep on memory, enabling information learned across different points in time to be integrated in a way that could not happen during retrieval practice.

### The role of temporal context during wake

Our modeling of the Liu and Ranganath (2021) task showed that the effects of retrieval practice on untested information during wake were determined by temporal context. The significance of temporal context in organizing episodic memory is well documented in the literature. For instance, in free recall tasks, participants commonly recall items that were presented close together during the learning phase (Healey et al., 2019; Howard & Kahana, 2002). In the model, during encoding, the hippocampus bound inputs about picture–word associations with entorhinal cortex (EC) activity patterns that gradually change over time (Howard & Kahana, 2002; Tsao et al., 2018). During retrieval practice, the hippocampus, triggered by the picture cue and list context, could recover the target pattern from the original activity (i.e., “pattern completion”; O’Reilly et al., 2014). Note that, in the close condition, both the cue and the list context overlapped between retrieval practice targets and nontargets that subjects were not instructed to retrieve. Our simulations revealed that, because of the overlap in cue and list context information, the hippocampus recovered information about both the target and the nontarget from the same list. The hippocampal output of the target and nontarget provided a training signal for the neocortex during retrieval practice (Liu et al., 2021). Specifically, learning in our model primarily relied on error-driving learning. When the relevant nontarget was retrieved with the target in the hippocampus, the diversity between the more accurate hippocampal representation and the less accurate neocortical created an error signal that trained the neocortical representation and facilitated later recall of both targets and nontargets.

In contrast to the near condition, in the far condition, nontargets were associated with different list contexts. As a result, during retrieval practice, only the target along with irrelevant noise could be recovered. In other words, the list context acted as a barrier that kept memories separated from each other. Thus, the diversity between retrieved irrelevant noise in the hippocampus and the neocortical representation also created an error signal that led to interference in the neocortex.

One of the key insights derived from our simulations is that retrieval-induced facilitation and forgetting can both emerge through changes in the neocortical representation—retrieval practice facilitates neocortical learning of nonpracticed associates from the same context and disrupts representations of nonpracticed associations from different contexts.

Our simulations also provide new insights into the relationship between retrieval-induced forgetting and retrieval-induced facilitation. Specifically, the dynamics of the model in the far condition align with theories that emphasize interference as a factor in retrieval-induced forgetting (e.g., Dodd et al., 2006; Jonker et al., 2013; C. M. MacLeod et al., 2003; Perfect et al., 2004; Raaijmakers & Jakab, 2013; Verde, 2012), and theories proposing that integration is a key factor in promoting retrieval-induced facilitation (Anderson & McCulloch, 1999; Chan, 2009). In both cases, temporal context determines whether retrieval practice will create interference or facilitate integration.

It is noteworthy that in the empirical data for the wake condition, semantic associations exert a less pronounced effect within the temporally close condition. This discrepancy may stem from the model’s assumption that temporal context and semantic associations operate independently, albeit in competition. In the wake condition, the model suggests that temporal context is the primary driver of memory integration, which may diminish the facilitative effects of semantic relatedness. Consequently, RIFA emerges when pairs are encoded within the same temporal context, and RIF is observed under more varied temporal contexts regardless of semantic relatedness.

However, the model might not fully replicate the nuanced dynamic observed between temporal context and semantic relationships in empirical studies, such as those reported by Liu and Ranganath (2021), where semantic relationships contribute to memory performance in the temporally close condition but not in the temporally far condition. This discrepancy underscores a limitation of the current model, which does not account for the potential interaction between temporal context and semantic associations.

### The role of temporal context during sleep

Our simulations with TEACH also revealed how sleep can mediate the effects of retrieval practice on untested information. As mentioned earlier, during wake, temporal context plays a key role in determining whether retrieval practice will increase competition or facilitate retrieval of nonpracticed items. Temporal context acted as a barrier during the wake condition, such that successful retrieval of the target pattern could spread to the nontarget sharing the same context, but retrieval did not extend across lists. In contrast, we simulated reactivation during sleep by deactivating the pools of temporal context. This was critical in accounting for the entire pattern of results, such that the impairment effects of retrieval practice observed during wakefulness shifted to facilitation following sleep. When temporal context input was deactivated during sleep, the result was a loss of this temporal context barrier, keeping memories separated from each other, which in turn enabled the cortical semantic layer to drive learning in the EC layer. Thus, far-related items benefitted from reactivation during sleep because reactivating a picture–word association could activate the semantically related word, which was also paired with the picture but encoded with different context input. In contrast, far-unrelated items did not benefit from sleep because input from the semantic layer was not sufficient to reactivate unrelated nontargets.

Our simulations suggest that the deactivation of temporal context input during sleep was critical for accounting for the full pattern of results. We think this assumption is a reasonable assumption for the following reasons: First, in addition to Liu and Ranganath (2021), this hypothesis is supported by recent studies showing that memory reorganization during sleep is predominantly driven by semantic relationships rather than temporal context (Schechtman et al., 2023a, 2023b).

Second, temporal context is usually assumed to reflect the outcome of a number of processes that support conscious, goal-directed activity, and context is widely believed to be used to cue recall of studied information (Polyn & Kahana, 2008). Presumably, it is unlikely that these processes would contribute to reactivation during sleep. For instance, in our case, participants encoded each pair in the context of a list of learned associations, and during retrieval practice, information about the list context could be intentionally used to cue retrieval. In contrast, reactivation during sleep is not driven by a current goal or task, and as such, it seems unlikely that context would be used to cue hippocampal reactivation during sleep.

The third factor to consider is that brain activity states fundamentally differ between wake and sleep. For instance, the PFC is engaged during active task performance, like memory encoding and retrieval practice, but evidence from multiple studies suggests that the PFC is deactivated during sleep (Hobson & Pace-Schott, 2002). For example, Braun et al. (1997) showed cerebral blood flow in the PFC was reduced during both slow-wave sleep and rapid eye movement (REM) sleep. The PFC has been widely implicated as an important source of temporal context that feeds into the hippocampus (Jenkins & Ranganath, 2010, 2016; Polyn & Kahana, 2008; Reeders et al., 2021). Thus, it is reasonable to think that, because PFC is deactivated during sleep, retrieval might be driven more heavily by semantic associations, rather than temporal context.

It is essential to acknowledge, however, that the assumption that temporal context is entirely dormant during sleep may not encapsulate the entirety of the sleep state’s complexity. While empirical evidence suggests that semantic connections may overshadow temporal context during sleep (Liu & Ranganath, 2021; Schechtman et al., 2023a, 2023b), it is improbable that the processing of temporal context is completely halted. Therefore, the model’s approach in deactivating temporal context input during sleep could be an oversimplification. This represents a limitation of the current model, and future research should strive to delineate a more precise delineation of the interplay between semantic and contextual associations in memory processing during sleep.

### Bidirectional interactions between the hippocampus and neocortex

Another key principle to emerge from the current model is that the effects of sleep on memory emerge from bidirectional interactions between the hippocampus and neocortex. Our model assumes that, during sleep, reactivation in the neocortex trains the hippocampus with both the directly reactivated target pattern from the input layer and the semantically related nontarget pattern generated from the neocortical semantic layer. In other words, reactivation during sleep enabled the neocortex to train the hippocampus to strengthen links between related pairmates, even if they were not learned in the same context. Pattern completion in the hippocampus, in turn, trained the cortical representation further. Put another way, our model suggests that the neocortex and hippocampus train each other during sleep, contrary to the unidirectional training from the hippocampus to the neocortex that characterizes retrieval practice.

Notably, this assumption diverges from the one-way relationship between the hippocampus and neocortex envisioned in the original CLS framework (McClelland et al., 1995; Singh et al., 2022). Specifically, McClelland et al.(1995) proposed that the hippocampus “teaches” the neocortex during sleep, and they simulated this effect by comparing the effects of fast learning and slow interleaved learning in connectionist models. Fast learning produced catastrophic interference (see also McCloskey & Cohen, 1989), and slow, interleaved learning allowed the new information to be readily learned without disrupting existing representations, such that the model could pick up the shared structure across most of the learned items.

Our model, in contrast, is based on recent evidence that memory consolidation involves bidirectional interactions between the hippocampus and neocortex. The dialogue between the hippocampus and neocortex may be initiated in the neocortex (Rothschild et al., 2017). In other words, reactivation in the neocortex may precede and trigger reactivation in the hippocampus. For example, prior studies found that reactivation in the visual cortex and auditory cortex preceded reactivation in the hippocampus and demonstrated that cortical reactivation patterns could predict subsequent hippocampal reactivation (Ji & Wilson, 2007).

### Retrieval practiced associations are prioritized for reactivation during sleep

Another important factor revealed in our simulations was the prioritized reactivation of retrieval practiced associations during sleep. Indeed, the switch from impairment to facilitation after sleep was not shown in the Sleep-Weak and Sleep-All models. We found that, if nonpracticed (i.e., weakly learned) associations were prioritized for reactivation or all previously learned associations were equivalently reactivated, the benefit of sleep in retrieval practiced associations was less or equal to control association. Thus, the impairment caused by retrieval practice could not be overcome during sleep.

We think that it is reasonable to assume that practiced associations are prioritized for reactivation during sleep, as it would be adaptive to preferentially retain experiences that are salient or important enough to be retrieved fairly often. However, this assumption might seem to be at odds with studies examining interactions between sleep and retrieval practice effects. For example, Bäuml et al. (2014) showed that, without feedback during retrieval practice, sleep produced larger benefits for restudied than retrieval practiced information (see also Antony & Paller, 2018). Thus, the testing effect was reduced after sleep. However, if corrective feedback was provided after retrieval practice, the benefit of sleep was comparable in restudied, and retrieval practiced items, eliminating the modulating role of sleep (Abel et al., 2019). This pattern suggests that the effect of sleep may be explained by the distribution-based bifurcation model of the testing effect (Halamish & Bjork, 2011; Kornell et al., 2011). Specifically, without feedback, retrieval practice strongly strengthens correctly retrieved items while leaving forgotten items unaffected, whereas restudy strengthens all restudied items to the same moderate degree. The strength of forgotten items may be too far from the retrieval threshold and cannot reach the threshold even with sleep. Therefore, on average, the sleep benefit is reduced in the retrieval practice condition. On the contrary, with feedback, the strength of forgotten items is raised above or close to the threshold, enabling these items to benefit from sleep. Therefore, the modulation role of sleep is eliminated.

### Relationship with prior findings on RIF and RIFA

While our goal is to provide a computational model accounting for the interactions between temporal and semantic relationships and sleep, as demonstrated in Liu and Ranganath (2021), our model also sheds light on the extensive literature on RIF and RIFA. The empirical study we modeled used a paradigm adapted from Jonker et al. (2018), which diverges from the typical RIF paradigms (e.g., Anderson et al., 1994). Specifically, the typical RIF paradigm uses associations between semantic categories and multiple exemplars (e.g., fruit–apple). Both Jonker et al. (2018) and Liu and Ranganath (2021) found RIFA when the two associations with the same cue were presented continuously, contrasting with the usual RIF observed in classical paradigms, where RIF is consistently reported unless participants are instructed to integrate the items.

We propose that the observed difference arises from several factors inherent in the designs of Jonker et al. (2018) and Liu and Ranganath (2021), which promote integration and reduce RIF. First, there were multiple cycles of study and retrieval practice in both studies. Prior studies have shown that reexposure to untested information after retrieval practice can reduce RIF (Storm et al., 2012). Second, in both studies, each scene was paired with only two objects, facilitating the formation of a coherent integrated imagination of two objects within the same scene, leading to RIFA.

Furthermore, our model addresses the varied findings on the effects of delay and sleep on untested information. While some studies report a reduction or elimination of RIF or even the emergence of RIFA following a long delay with sleep (e.g., Abel & Bäuml, 2014; Baran et al., 2010; Chan, 2009; MacLeod & Macrae, 2001; Oliva & Storm, 2023), others observed that RIF persists after sleep but is eliminated after a delay without sleep (Abel & Bäuml, 2012; Racsmány et al., 2010).

Liu and Ranganath (2021) showed both decreased RIF (switching to RIFA) and increased RIF following sleep compared with awake, as a function of semantic relationships between the target and nontarget. Our current model shows that the key factor moderating the effect of sleep is whether targets and nontargets can be integrated through semantic relationships. This finding is consistent with the literature where studies showing RIFA following sleep used materials that are easy to integrate, such as prose passages. Future empirical studies need to systematically manipulate the degree of integrability to determine the boundary conditions that affect the consequences of sleep.

### Future directions

It is noteworthy that other mechanisms may also potentially account for the integration of related information during sleep. For example, Singh et al. (2022) simulated the alternation between SWS, during which the hippocampus trained the neocortex with newly acquired memory, and REM sleep, during which the neocortex primarily replayed the consolidated remote memory without influence from the hippocampus. Therefore, it is possible that the replay of semantic connections during REM sleep directly overwrites the impairment of nontargets caused by retrieval practice and leads to facilitation. However, the switch from impairment to facilitation is less likely to be hippocampal-independent and exclusively driven by REM sleep. If so, the model would need to prioritize REM sleep extensively to overcome the interference created by the hippocampus. Future modeling work may incorporate the alternation between SWS and REM sleep. Future neuroimaging work may test whether the integration of related information during sleep involves both the hippocampus and neocortex.

## Conclusion

In summary, the current model provides a compelling mechanistic explanation for why we might integrate memories overnight. Moreover, this model offers some interesting new examples of the diverse ways in which the cortex and hippocampus can interact during learning. During retrieval practice, the hippocampus effectively trains the cortex through its ability to pattern complete the associated word and drive activation of that word in the cortex, causing a small but behaviorally significant impact on the cortical connections between the word and the picture (Liu et al., 2021). However, during sleep, the roles are reversed. The semantic connections among the words allow the cortex to train the hippocampus to learn about this connection effectively. This case also suggests that the hippocampus may not always engage in pattern separation to keep memories separate. However, it may also learn systematic connections among different memories, providing a precursor to further slow semantic learning in the cortex, as envisioned in the standard CLS model. Thus, these systems may work together synergistically while still having very different biases in the way they learn overall. Finally, this work provides a novel way of understanding the conditions under which retrieval-induced forgetting versus retrieval-induced facilitation should be observed (Liu et al., 2021; Liu & Ranganath, 2021), according to the overall dynamics of representational patterns being pushed further apart vs. integrated together.

## Open practices statement

Upon publication, the full implemented model and data can be downloaded: https://github.com/ccnlab/teach-sleep
